# Photo-Printing in Colors

**Published:** 1882-05

**Authors:** 


### PHOTO PRINTING IN COLORS.

Some specimens of color printing in Herr Albert's studio deserve mention. Herr Albert has been experimenting with some success on the method which is usually connected with the name of Ducos du

Hauron. A painting is photographed three times ; the first negative is taken through a red screen, the second through a blue screen, and the third through a yellow screen. Albert employs colored liquids for his screens, and in this way he secures three negatives, in the taking of which respectively the rays of the three primary colors have been absorbed. The negative taken through the red screen is then printed upon red carbon tissue, and the other two negatives printed respectively with blue and yellow tissue. Then the three prints in red, yellow, and blue are superposed, and the picture is finished. By working in this way, Herr Albert claims to have reproduced a colored picture of Lemercier, which had been produced from eighteen stones (and therefore contained eighteen different tints), in all its pristine beauty. As the original was not at hand for comparison, it was impossible to say how far success had been secured, but certainly the Albert pictures are very pleasing and interesting. To talk of the process as one of photographing in natural colors is, of course, nonsense, for there is no relation between the colors absorbed by the screens and the particular pigment which happens to be in the carbon tissue ; but, as an important experiment, the matter deserves notice. But more satisfactory still than the compound pictures in colors, was a compound picture we saw (from the three negatives) printed in monochrome or sepia ; the painting was here reproduced far more satisfactorily than when a single negative only was employed for printing. In effect, the colors of the original picture appeared to have been more truthfully translated into monotone by taking three negatives under the particular conditions ; so harmonious, indeed, was the result, that we should not be surprised if in future, in the photographing of paintings, recourse is not had to this particular method, or a modification of it.—*Photo News.*

